# Development of a test that measures real-time HER2 signaling function in live breast cancer cell lines and primary cells

**DOI:** 10.1186/s12885-017-3181-0

**Published:** 2017-03-16

**Authors:** Yao Huang, David J. Burns, Benjamin E. Rich, Ian A. MacNeil, Abhijit Dandapat, Sajjad M. Soltani, Samantha Myhre, Brian F. Sullivan, Carol A. Lange, Leo T. Furcht, Lance G. Laing

**Affiliations:** 1 Celcuity LLC, Minneapolis, MN USA; 2 Division of Hematology, Oncology, and Transplantation, Departments of Medicine and Pharmacology and The Masonic Cancer Center, University of Minnesota, Minneapolis, MN USA; 3 Department of Laboratory Medicine and Pathology, University of Minnesota, Minneapolis, MN USA

**Keywords:** CELx HSF Test, Cancer diagnostic, HER2-negative, HER2-positive, Breast cancer, Signaling pathway, Targeted therapeutics, Oncology, Breast tumor, Primary epithelial cells

## Abstract

**Background:**

Approximately 18–20% of all human breast cancers have overexpressed human epidermal growth factor receptor 2 (HER2). Standard clinical practice is to treat only overexpressed HER2 (HER2+) cancers with targeted anti-HER2 therapies. However, recent analyses of clinical trial data have found evidence that HER2-targeted therapies may benefit a sub-group of breast cancer patients with non-overexpressed HER2. This suggests that measurement of other biological factors associated with HER2 cancer, such as HER2 signaling pathway activity, should be considered as an alternative means of identifying patients eligible for HER2 therapies.

**Methods:**

A new biosensor-based test (CELx^TM^ HSF) that measures HER2 signaling activity in live cells is demonstrated using a set of 19 human HER2+ and HER2– breast cancer reference cell lines and primary cell samples derived from two fresh patient tumor specimens. Pathway signaling is elucidated by use of highly specific agonists and antagonists. The test method relies upon well-established phenotypic, adhesion-related, impedance changes detected by the biosensor.

**Results:**

The analytical sensitivity and analyte specificity of this method was demonstrated using ligands with high affinity and specificity for HER1 and HER3. The HER2-driven signaling quantified ranged 50-fold between the lowest and highest cell lines. The HER2+ cell lines were almost equally divided into high and low signaling test result groups, suggesting that little correlation exists between HER2 protein expression and HER2 signaling level. Unexpectedly, the highest HER2-driven signaling level recorded was with a HER2– cell line.

**Conclusions:**

Measurement of HER2 signaling activity in the tumor cells of breast cancer patients is a feasible approach to explore as a biomarker to identify HER2-driven cancers not currently diagnosable with genomic techniques. The wide range of HER2-driven signaling levels measured suggests it may be possible to make a distinction between normal and abnormal levels of activity. Analytical validation studies and clinical trials treating HER2- patients with abnormal HER2-driven signaling would be required to evaluate the analytical and clinical validity of using this functional biomarker as a diagnostic test to select patients for treatment with HER2 targeted therapy. In clinical practice, this method would require patient specimens be delivered to and tested in a central lab.

**Electronic supplementary material:**

The online version of this article (doi:10.1186/s12885-017-3181-0) contains supplementary material, which is available to authorized users.

## Background

Molecularly targeted therapies represent a major advance in cancer treatment. Amongst the most consequential therapies are those targeting human epidermal growth factor receptor 2 (HER2). HER2 overexpression or gene amplification is associated with more aggressive disease progression, metastasis, and a poor clinical prognosis in breast and gastric cancer [1, 2]. Current FDA-approved treatments for HER2 overexpressed or gene amplified (HER2+) breast cancers have significantly improved clinical outcomes in the metastatic and adjuvant settings and include small-molecule kinase inhibitors, such as lapatinib (Tykerb), monoclonal antibodies, such as trastuzumab (Herceptin) and pertuzumab (Perjeta), and antibody-drug conjugates, such as ado-trastuzumab emtansine (Kadcyla) [2, 3].

The conventional opinion that only patients with HER2+ tumors benefit from HER2-targeted therapies has been questioned by the review of results from several studies and trials. While clinical trials conducted specifically to evaluate the efficacy of different HER2 therapies in HER2– patients have largely generated negative overall results, some have suggested that a sub-group of HER2- patients benefited. In one trial, estrogen receptor-positive (ER+)/HER2- patients who entered the study with a median of less than one month since discontinuation of tamoxifen showed a statistically nonsignificant trend toward improvement in both progression free survival and clinical benefit rates that was nearly identical to that found in a group of ER+/HER2+ patients [4]. In another trial involving HER2- breast cancer patients, treatment with lapatinib led to a statistically significant 27% downregulation of Ki67 [5]. In this same trial, 14% of HER2-negative patients showed a >50% reduction in Ki67 suggesting the existence of a responding subset of the HER2– population. Finally, re-analyses of previous trials indicate no significant correlation exists between *HER2* gene copy number and trastuzumab benefit and that a sub-group of HER2- breast cancer patients inadvertently included in a trial intended for HER2+ patients benefited from HER2-targeted therapies [6–9].

These results highlight the challenge of identifying a targeted therapy benefit in HER2-breast cancer patients when only a sub-group of 10–20% of them may be responsive. No genomic-derived biomarker correlates for this sub-group have been discovered. This suggests that another biological factor associated with HER2 cancer, dysfunctional HER2-driven signaling, may be a potential diagnostic factor to consider as an alternative to measurement of HER2 expression levels.

HER2 belongs to the human epidermal growth factor receptor (HER) family of receptor tyrosine kinases, which also includes HER1 (known as epidermal growth factor receptor (EGFR)), HER3, and HER4. The HER family members are expressed in many tissue types and play a key role in cell proliferation and differentiation. The HER receptors are generally activated by ligand binding leading to the formation of homo and heterodimers followed by phosphorylation of specific tyrosines in the cytoplasmic domain. In the HER family signaling system, EGF specifically binds to EGFR, and NRG1b specifically binds to HER3 and HER4. HER1 and HER4 are fully functional receptor tyrosine kinases, whereas HER2 has no endogenous ligand and HER3 has a weakly functional kinase domain. Due to the absence of a specific ligand for HER2, HER2 primarily functions as a ligand dependent heterodimer with other members of the HER family [10]. The combination of receptor dimers influences subsequent signaling pathways. For example, the HER1/HER2 heterodimer mainly activates the Ras/MEK/ERK (MAPK), and PI3K/Akt signaling pathways [11]. Increasing evidence suggests that HER3 is the preferred partner and to a somewhat lesser extent EGFR and HER4 for amplified HER2 in breast cancer [12–14]. The HER2/HER3 heterodimer relies on HER3 for its signaling, and HER3 can bind to p85 and strongly activate the PI3K/Akt pathway [14, 15]. In addition, Hendriks et al. has proposed that activation of ERK (MAPK) by HER2 arises predominantly from HER1/HER2 heterodimers using their study models [16]. Ligand binding triggers scaffolding formation and downstream signaling cascades by recruitment of specific substrate proteins [10]. Finally, other work has demonstrated ~10^7^ different states for HER1 that have very rapid dynamics. Assuming that this accounting could be applied to the other very similar receptors in the HER family, this may explain why proteomic methods may be unable to appropriately measure HER family-initiated signaling dysfunction [17].

Label-free biosensor assays can provide real-time measurement of cellular responses without the limitations of standard endpoint assays. A biosensor is an analytical platform that uses the specificity of a biological molecule or cell along with a physicochemical transducer to convert a biological response to a measureable optical or electrical signal. A class of biosensor-based, label-free, whole-cell screening assays offers an unprecedented combination of label-free detection with sensitivity to live-cell responses and has emerged as an useful tool in high-throughput screening (HTS) for the discovery of new drugs over the past years [18]. Label-free whole-cell assays offer a number of advantages. Most importantly, biosensors can directly measure inherent morphological and adherent characteristics of the cell as a physiologically or pathologically relevant and quantitative readout of cellular response to signaling pathway perturbation. Numerous research groups have demonstrated that biosensor-based cell assays can quantitatively monitor dynamic changes in cellular features such as cell adhesion and morphology for complex endpoints that are modulated by many signal transduction pathways in live adherent cells [19–21].

The potential of biosensor-based, label-free, whole-cell assays to accurately identify pathway-driven disease and reliably serve as clinical diagnostic tools remains to be explored. The current work represents the first feasibility assessment of viable cell signaling from cell lines and primary cells in real time by applying a cell biosensor assay methodology. The focus of this study is on the HER2 signaling pathway in breast cancer using an impedance whole-cell biosensor with well-established reference breast cancer cell lines. Results for a feasible and reliable biosensor-based label-free assay, the CELx HER2 Signaling Function (HSF) test, are presented to accurately determine whether live cells have abnormally amplified HER2 pathway signaling activities and how the pathway responds to HER2-targeted drugs *in vitro*. As a proof-of-concept for potential clinical applications, the test is applied to two patient tumor specimen-derived primary cell samples *ex vivo*.

## Methods

### Chemicals and reagents

Recombinant human epidermal growth factor (EGF), neuregulin 1b (NRG1b), and insulin like growth factor-1 (IGF-1) were purchased from R&D Systems (Minneapolis, MN). Collagen was obtained from Advanced Biomatrix (Carlsbad, CA) and fibronectin was obtained from Sigma (St. Louis, MO). Lapatinib, afatinib, linsitinib, GSK1059615, trametinib, doramapimod, and SP600125 were purchased from SelleckChem (Houston, TX) and prepared at stock concentrations in fresh 100% DMSO before final dilution into assay medium. Pertuzumab was obtained from Kronan Pharmacy (Uppsala, Sweden).

### Cell culture

Human breast cancer cell lines used in this study included SKBr3, BT474, BT483, T47D, MCF-7, AU565, CAMA1, ZR75-1, ZR75-30, HCC202, HCC1428, HCC1569, HCC1954, MDA-MB134vi, MDA-MB175vii, MDA-MB231, MDA-MB361, MDA-MB415, MDA-MB453 (all from ATCC, Manassas, VA), and EFM192A (from Leibniz Institute DSMZ, Germany). All cell media were from Mediatech (Manassas, VA) and fetal bovine serum (FBS) was from Hyclone (Logan, UT). AU565, ZR75-1, ZR75-30, HCC202, HCC1428, HCC1569, HCC1954, and EFM192A were maintained in RPMI 1640 containing 10% FBS. T47D and BT483 were maintained in RPMI 1640 containing 10% FBS and 10ug/mL human insulin (Mediatech, Manassas, VA). MDA-MB134vi, MDA-MB175vii, MDA-MB231, MDA-MB361, and MDA-MB453 were maintained in DMEM containing 10% FBS. MDA-MB415 was maintained in DMEM containing 15% FBS, 10ug/mL human insulin, and 10ug/mL glutathione (Sigma, St. Louis, MO). BT474 and CAMA1 were maintained in EMEM containing 10% FBS. MCF-7 was maintained in EMEM containing 10% FBS and 10ug/mL human insulin. SKBr3 was maintained in McCoy’s containing 10% FBS. The cell lines were authenticated in March 2016, by ATCC, and results were compared with the ATCC short-tandem repeat (STR) database.

The use of excess surgically resected human breast cancer tissue in this study was received from the University of Minnesota tissue procurement department (Minneapolis, MN) and Capitol Biosciences tissue procurement services (Rockville, MD). The material received was excess tissue and de-identified. Liberty IRB (Columbia, MD) determined that this research does not involve human subjects as defined under 45 CFR 46.102(f) and granted exemption in written form. The data were analyzed and reported anonymously. Patient specimens were received from the clinic at 0–8 °C within 24 h from removal. Methods for tissue extraction, primary cell culture, and short-term population doublings are essentially as described previously [22, 23]. Briefly, 20–70 mg tissue was minced with scalpels to <2 mm pieces and cryopreserved until testing [24] or used fresh. Tissue (20–40 mg) for CELx HSF testing was enzymatically disaggregated for minimal time to obtain cells and cell clusters in collagenase and hyaluronidase (Worthington Biochemical, Lakewood, NJ) at 37 °C in 5% CO_2_. On the same day as digestion, the disaggregated tissue was washed in culture media to remove disaggregation enzymes, plated on 6-well tissue culture plates in serum-free mammary epithelial cell media, and grown 4–14 days until approximately 2 × 10^5^ cells were available. Trypan blue staining was used before initial plating to determine the viability of each specimen.

### Real-time assessment of HER2 signaling network activity

Experiments were performed using the xCELLigence Real Time Cell Analyzer (RTCA) (ACEA Biosciences, San Diego, CA), an impedance-based biosensor, which was placed in a humidified incubator at 37 °C and 5% CO_2_. Cells were seeded in triplicate in 96-well sensor plates (pre-coated with collagen and fibronectin) in serum-free minimal medium (assay medium) the day before ligands were added. The impedance CI value reflects the aggregate of cellular events that include the viability of the cells, the relative density of cells over the electrode surface, morphological changes, and the relative adherence of the cells. The adherence characteristic is dependent on the type and concentration of adhesion proteins on the cell surface and is regulated at least in part by cellular signaling through cell-cell and cell-ECM interactions. Automatic impedance recording began after cell seeding and continued throughout the whole course of an experiment, ending 6–10 h after growth factor addition. The instrument software converts impedance in ohms (Ω) into a cell index (CI) value by the algorithm CI = Ω/15. In the case of drug/inhibitor pretreatment, drugs/inhibitors were freshly prepared in assay medium at 20× of working concentrations and added into the sensor plates two hours prior to the addition of growth factors.

To ensure dynamic pathway signaling related events are the primary cell activity measured, and that the effect of cell proliferation is excluded, only CI values collected within 30 h of seeding were analyzed in the CELx HSF test. This 30-h period includes the time just after the cells are seeded onto the sensor up to the time point 6–10 h after growth factor addition. The signaling activity following growth factor addition is the only relevant time period for the CELx test measurand as it corresponds to the period when dynamic pathway signaling is occurring in the cell sample.

In the CELx HSF test feasibility work described herein, EGF or NRG1b stimulation was used in combination with specific types of HER2 inhibitors to provide insights into dimerization of HER2 related to CELx Test signals. Growth factors were freshly prepared in the same assay medium at 10X of working concentrations and added 18–24 h after cell seeding. The same volume of assay medium instead of the growth factors/drugs/inhibitors was added in the “blank”, media only wells (control wells). All additions were performed with a VIAFLO automatic liquid handler (Integra Biosciences, Hudson, NH).

Two inhibitory molecules were selected that act by directly binding the receptor and affecting signaling initiation. Lapatinib is a small-molecule kinase inhibitor that blocks receptor signaling processes by reversibly binding to the ATP-binding pocket of the protein kinase domain of HER family members, preventing receptor phosphorylation and activation [25]. Pertuzumab is an anti-HER2 mAb that inhibits dimerization of HER2 with other receptors by binding to subdomain II of the HER2 protein and has been shown to interfere with HER2 signaling [26, 27].

### Data analysis and statistics

CELx test data was exported from the RTCA software file for the time versus Cell Index (CI) analysis by TraceDrawer (Ridgeview Instruments, Sweden) and Microsoft Excel. The cell index versus time course data essentially fell into one of 3 groups for each cell sample tested: cells with addition of media only (C), cells with addition of growth factor stimulus only (CF), and cells with addition of an antagonist drug followed by a growth factor stimulus (CDF). To permit inter-sample quantitative comparison, the cell index was set to zero for each set of CI versus time course data at the time point of stimulus addition to a cell sample. After the stimulus was added, data were assessed using the CI versus time data by one of the following algorithms:

- For determining the magnitude of the stimulus, CF-C was used.
- For determining the absolute amount of HER2 involvement in a particular stimulus in the CELx HSF test, (CF-C)-(CDF-C) was used, combining the EGF and NRG1b stimulus data to arrive at a comparative total amount of HER2 signaling response for a particular cell sample.
- Percentage of stimulus signal reduction by drug inhibition was calculated by [1-[(CF-C)-(CDF-C)]/ (CF-C)]*100.

All dose–response curves were obtained using nonlinear regression curve fitting with GraphPad Prism 6 (GraphPad Software, La Jolla, CA). Pearson correlation analysis was performed using GraphPad Prism 6 to evaluate the relationships among the variables of interest. *P* < 0.05 was considered statistically significant.

### Flow cytometry (fluorescence-activated cell marker analysis)

Flow cytometric analysis of luminal (EpCAM^+^, Claudin4^+^) and basal (CD49f^+^, CD10^+^) markers as well as estrogen receptor (ER) and progesterone receptor (PR) was performed on the primary samples to confirm epithelial cell identity and that fibroblast content was low. Fluorescence flow cytometry was also used to assess protein expression levels of the cell lines and primary cells used in this study. Antibodies used in this study are described in Additional file 1: Table S1. Sample data was collected on a BD FACSCalibur (BD Biosciences, San Jose, CA) equipped with a 488-nm and 637-nm laser. Data were analyzed with FlowJo 2 (FlowJo LLC, Ashland, OR).

## Results

### Basic principle of the CELx HER2 signaling function test for real-time assessment of the HER2 signaling network

**Fig. 1:**
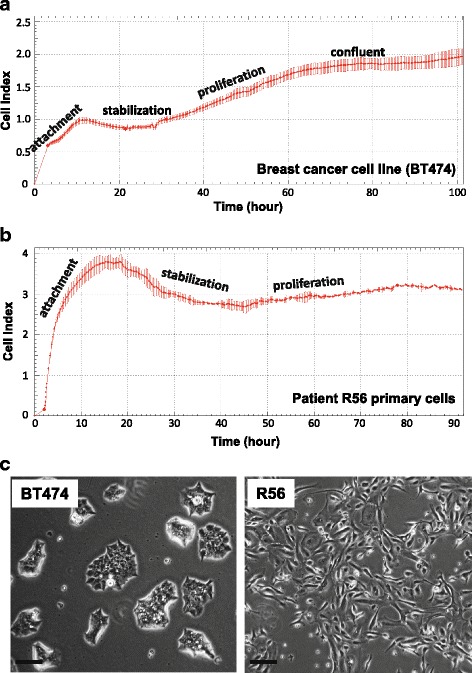
Representative CI versus time-course curves for basic cell attachment. Human breast cancer BT474 cells (**a**) or R56 patient-derived primary breast tumor cells (**b**) were seeded in a sensor plate and allowed to adhere, spread, and proliferate. Impedance was recorded as Cell Index (CI) versus time for 100 h after seeding. Cell attachment, stabilization, proliferation, and confluent phases are shown as indicated. **c** Representative images captured by an inverted phase contrast microscope (magnification: X40) showing cell morphology of BT474 and breast cancer R56 primary cells. Scale bar, 100 μm

One of the first properties noted with the biosensor performance was that absolute baseline attachment CI values can be variable among different reference cell lines derived from the same tissue type. This could be influenced by cell morphology and the exact nature of cell attachment. Cells from the same sample gave very similar well-to-well CI values for baseline attachment. We found no significant correlation between this baseline attachment impedance and the magnitude of the signaling response upon cell perturbations. Using the human breast cancer BT474 cell line as an example, a typical CI time-course curve of over approximately 100-h period after seeding onto the sensor plate is shown, including quantitative measurement of initial cell attachment (~1CI, about ~200x background of 0.005CI), reflecting the balance of settling, adhesion, spreading), lag (plateau and stabilization), logarithmic growth (proliferation), and formation of a cell (mono) layer (Fig. 1a). Human breast tumor-derived primary cells displayed a similar CI time-course curve and a representative curve of patient R56 primary cells is shown in Fig. 1b. The initial cell adhesion (<20 h, 3.8CI) CI is somewhat higher, whereas the cell proliferation slope is similar compared to other breast cancer cell lines; though the slope of cells from different specimen can vary depending on the disease state. These observations are consistent with morphology differences (Fig. 1c) and the cell proliferation rates. The baseline attachment additionally serves as a quality control that live cells are being applied to the assay vessel before any other assay steps are performed.

Cell seeding density is a critical factor in establishing a useful dynamic range for CI values that encompass the spectrum of attachment values observed using different cell lines. The results indicated that 12,500 to 15,000 cells per well in a 96-well format sensor plate is the ideal seeding density, allowing cell-cell contacts that are required for authentic epithelial cell signaling. No significantly proportional increase in CI values was seen when higher densities of cells (>15,000 cells per well) were used. Thus, a seeding density of 15,000 cells per well provided a balance between signal magnitude and cell conservation when considering data from numerous breast cancer cell lines and primary cells.

### Pathway signaling measurement by the CELx HSF test

**Fig. 2:**
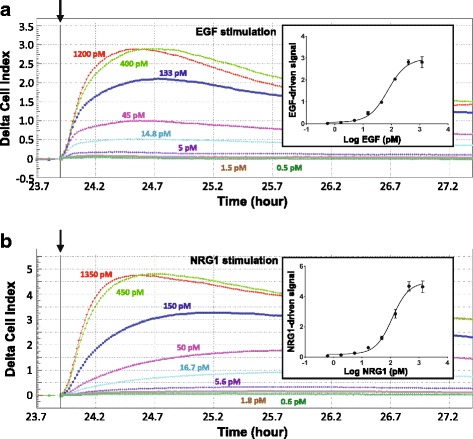
Dose–response curves of EGF and NRG1b stimulation of HER2 signaling in SKBr3 cells. SKBr3 cells were seeded in the sensor plates and stimulated with serial titrations of **a** EGF (0 pM to 1200 pM) or **b** NRG1b (0 pM to 1350 pM). Instrument data for CELx curves are displayed using Delta CI values to demonstrate the relative signals to the time point (*arrow*) when the stimulus (EGF or NRG1b) was added. Log plots of dose-response curves with error bars of EGF and NRG1b stimulation are shown in the insets for **a** and **b**, respectively﻿

SKBr3 HER2+ breast cancer cells in different wells of the 96-well biosensor were stimulated with EGF or NRG1b. Representative dose–response curves for EGF or NRG1b stimulation of SKBr3 HER2+ breast cancer cells are shown in Fig. 2. EGF and NRG1b activated the HER2 pathway by initially increasing the impedance values in a ligand concentration-dependent manner. The measured EC_50_ for EGF is 74.1 pM (Fig. 2a), with a 95% confidence range 62.08–88.44 pM. The measured EC_50_ for NRG1 is 114.7 pM (Fig. 2b), with a 95% confidence range 93.30–141.1 pM. In addition, both EGF and NRG1b signals peaked at stimulus dose of 400 pM to 800 pM. This peak dose range was also seen in other breast cancer cell lines.

### Pathway specificity and selectivity

**Fig. 3:**
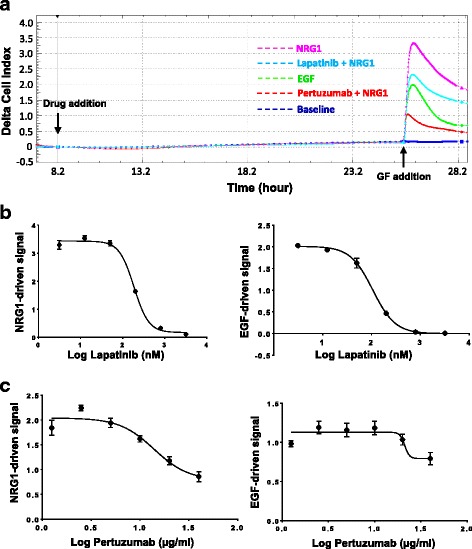
Dose–response curves showing the effects of HER2 inhibitors on EGF- and NRG1b-directed HER2 signaling. **a** Neither pertuzumab nor lapatinib has significant effect on baseline cell signal determined before agonist addition. SKBr3 cells were seeded in sensor plates and treated with pertuzumab (10 μg/mL), lapatinib (200nM), or vehicle (control) 18 h prior to stimulation with NRG1 or EGF. CELx curves are displayed using Delta CI values to easily compare the relative change in signals from the time point of drug addition. The time points for drug addition and growth factor (GF) addition are indicated by *black arrows*. **b** and **c** SKBr3 cells were seeded in sensor plates and treated with serial titrations of lapatinib (0 nM to 3200 nM) or pertuzumab (0 μg/mL to 40 μg/mL) two hours prior to stimulation with EGF or NRG1b. Dose–response curves of drug inhibition on NRG1b and EGF-driven cell index signals are displayed

To address whether pertuzumab and lapatinib have effects on the cells apart from inhibiting ligand-dependent HER2 activities, SKBr3 cells were pretreated with pertuzumab (10 μg/mL), lapatinib (200nM), or vehicle (control buffer) 18 h prior to stimulation with growth factors (NRG1 or EGR). As shown in Fig. 3a, during the 18-h drug treatment period (time points from drug addition to GF addition), there was no apparent difference in CELx test curves between untreated cells (control media only) and cells treated with pertuzumab or lapatinib. In contrast, both drugs exhibited significantly inhibitory effects on HER2 ligand (NRG1)-induced HER2 activities (see Fig. 3a, time points after GF addition). Dose–response curves are shown for lapatinib and pertuzumab inhibition with EGF and NRG1b stimulation, respectively, in SKBr3 cells (Fig. 3b and c). Lapatinib inhibited both EGF- and NRG1b-driven HER2 signals to the same level in SKBr3 (IC_50_ = 97nM for EGF-driven signal and IC_50_ = 175nM for NRG1b-driven signal) (Fig. 3b). In contrast, pertuzumab showed partial inhibition of both NRG1 and EGF with significantly higher levels of inhibition on NRG1b-driven signal than it did on EGF-driven signal (Fig. 3c). The measured IC_50_ for pertuzumab on NRG1 in SKBr3 is 13.94 μg/mL (Fig. 3b), with a 95% confidence range 9.21–21.02 μg/mL. Together, these findings demonstrated that the CI values measured indeed resulted from changes in the status of NRG1b- and EGF-elicited HER2 signaling activities. In most cell lines tested herein, a lapatinib concentration of 200nM showed the greatest inhibitory effect in sensitive cell lines while differentiating less sensitive cell samples. Pertuzumab was initially tested at a range of concentrations to determine the most effective concentration and then employed at a single maximal dose of 10 μg/mL for the remainder of the cell samples. Thus, 200nM of lapatinib and 10 μg/mL of pertuzumab were chosen as the doses to be used in these experiments.

A panel of pharmacological inhibitors that specifically inhibit different points in the PI3K and MAPK pathways was tested in order to determine which pathway(s) was critically involved in NRG1b- and EGF-directed HER2 signals in breast cancer and thereby specific cellular responses in our CELx HSF tests.

**Fig. 4:**
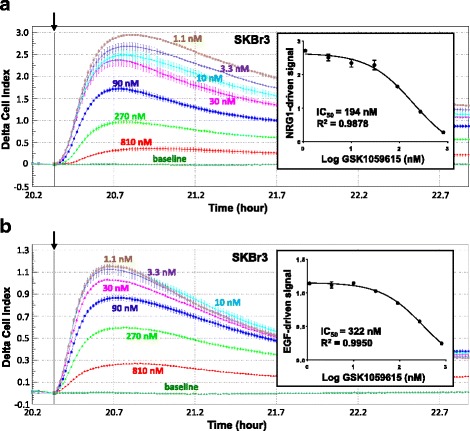
The PI3K/AKT pathway significantly contributes to the ligand-driven HER2 signaling activities detected by CELx HSF tests. **a** and **b** SKBr3 cells were seeded in sensor plates and then treated with a serial titration of the PI3K/AKT pathway inhibitor GSK1059615 (0 nM to 810 nM) two hours prior to maximal stimulation with NRG1b (800 pM) (**a**) or EGF (600 pM) (**b**). CELx curves are displayed using Delta CI values to demonstrate the relative signals to the time point (*arrow*) when the stimulus (EGF or NRG1b) was added. Dose–response curves of GSK1059615 inhibition on NRG1b and EGF-driven HER2 signals are shown in the *insets*

Dose–response curves of inhibitory effects of GSK1059615, a selective PI3K inhibitor [28], on ligand-driven HER2 signals were obtained in SKBr3 cells (Fig. 4a-b). These data demonstrated that inhibition of PI3K significantly reduced both EGF- and NRG1b-directed HER2 signals detected by CELx HSF tests in a drug dose-dependent manner. Similar results were obtained in other cell lines and with GDC-0941 [29], another selective inhibitor of PI3K (Additional file 2: Figure S1).

Trametinib, a specific inhibitor of MEK1/2, was also tested for the effect on inhibition of the MEK/ERK pathway on ligand-driven HER2 signals [30]. The results indicated that trametinib did not appear to have an inhibitory effect on either EGF- or NRG1b-driven HER2 signals or attenuate the impedance signal (Additional file 3: Figure S2) for these cell lines. Inhibition of the p38 MAPK pathway by doramapimod [31] (Additional file 4: Figure S3) or inhibition of the JNK pathway by SP600125 [32] (Additional file 5: Figure S4) had no significant impact on ligand-driven HER2 signals in the CELx HSF tests. Similar to what was observed with the MEK/ERK pathway inhibitor, the results with these inhibitors suggested that neither of these MAPK-associated pathways significantly contributed to the ligand-driven HER2 signaling activities detected in our CELx HSF tests of breast cancer cells.

### Cross-functional receptor specificity

**Fig. 5:**
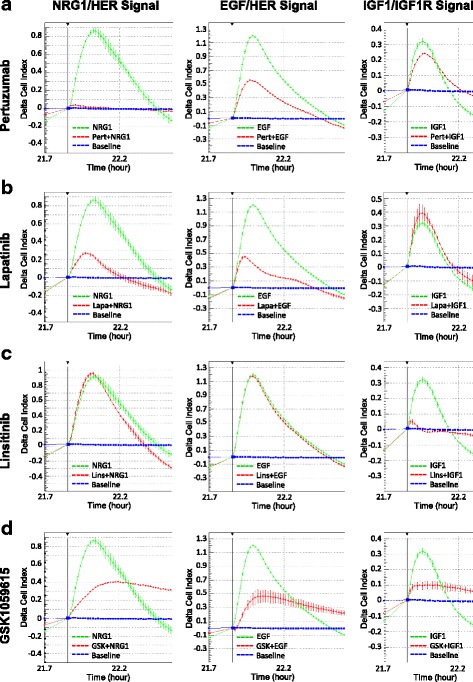
Comparison of EGF–HER2, NRG1b–HER2, and IGF-1–IGF-1R signaling systems in CELx assays. Human breast cancer T47D cells pre-seeded in sensor plates were treated with (**a**) pertuzumab (10 μg/mL), (**b**) lapatinib (200 nM), (**c**) linsitinib (200 nM), or (**d**) GSK1059615 (300 nM) two hours prior to stimulation with NRG1b (800 pM), EGF (600 pM), or IGF-1 (8 nM). CELx curves are displayed using Delta CI values to demonstrate the relative signals to the time point (*arrow*) when the stimulus (NRG1b, EGF, or IGF-1) was added. *Blue* curves, unstimulated cells (baseline); *Green* curves, cells stimulated with ligand (NRG1b, EGF, or IGF-1); *Red* curves, cells stimulated with ligand in the presence of drug (pertuzumab, lapatinib, linsitinib, or GSK1059615)

Growth factor receptor / receptor tyrosine kinase (RTK) signaling networks share many common features, such as interactions among ligands, antagonists (receptor inhibitors), and RTKs, receptor phosphorylation / activation, and activation of downstream pathways. All these factors could contribute to the CELx signals. Verification of the specificity and selectivity of the CELx HSF test was performed by evaluating whether the test response identifies solely HER2-related activity when HER family ligands are applied to the test cells. Additionally, testing was performed to determine whether the activity of antagonists at HER family receptors affects growth factor activity on other receptors and whether antagonists applied to other receptors affected growth factor activity on HER family receptors during the test. For an example of evaluating CELx for receptor cross-talk, the network profile of HER2 signaling was compared with that of insulin-like growth factor 1 receptor (IGF-1R) by utilizing specific agonists and antagonists for IGF-1R in the CELx assays. Using the T47D breast cancer cell line, IGF-1 induced substantial CELx signals through IGF-1R with an average Delta CI of 0.4 (Fig. 5, right panels). Comparing the magnitude of IGF-1/IGF-1R signals, NRG1b- and EGF-induced HER2 signals were much larger in these cells (Delta CI = 0.8 to 1.2; Fig. 5, left and middle panels). As expected, both pertuzumab and lapatinib significantly inhibited EGF- and NRG1b-driven HER2-related signals and had no effect on IGF-1–driven IGF-1R signals in CELx assays. In further evidence of the specificity of the test response, the IGF-1R kinase inhibitor, linsitinib [33], completely inhibited IGF-1-driven IGF-1R signals, but had no effect on either EGF or NRG1b-driven HER2 signals (Fig. 5c). As an additional control, GSK1059615, which specifically inhibits PI3K, the common effector downstream of two HER receptors and IGF-1R, significantly blocked all three ligand-receptor biosensor signals (Fig. 5d).

### Relating the magnitude of CELx HSF test signals to abnormal HER2 signaling activities in breast cancer cell lines

After confirming the selectivity and specificity of the CELx HSF test, ligand-driven HER2 signals were surveyed in 10 human breast cancer cell lines overexpressing HER2 (HER2+) and 10 human breast cancer cell lines expressing lower or normal levels of HER2 (HER2-) in order to determine whether CELx HSF test positive (HSF+) and CELx HSF test negative (HSF-) populations exist among HER2+ and HER2- cell types. These cell lines were chosen based on *HER2* gene expression recorded in public databases such as the Cancer Cell Line Encyclopedia (CCLE) [34]. Here an analysis is provided for the HER2 protein expression by fluorescence flow cytometry in all 20 cell lines at the time when cells were processed for CELx HSF tests. The flow cytometry dataset on HER2 expression status is consistent with the CCLE reference data (Additional file 6: Table S2). Two CCLE-listed HER2+ cell lines, MDA-MB453 and MDA-MB361, had much lower HER2 expression (approx. 500 mean fluorescence channel units (MFC)) than the HER2+ clinical standard control cell line, SKBr3 (2386 MFC). Consulting the CCLE gene copy number database for these two cell lines revealed that MDA-MB453 had normal *HER2* gene copy number and MDA-MB361 had more than 2.2 copies per cell. Another recent study indicated that MDA-MB361 had amplified gene copy number and would qualify as a clinical HER2+ [35]. The HER2 protein expression levels in the flow cytometry dataset placed both MDA-MB453 and MDA-MB361 in a lower range more closely associated with the HER2– group (Additional file 6: Table S2). Thus, these cell lines were considered according to their clinical assignment: MDA-MB453 is part of the HER2– group and MDA-MB361 is a member of the HER2+ group. One HER2- cell line (MDA-MB-134vi) was excluded from further analysis because it did not meet the CELx HSF test criteria for minimum baseline cell attachment on the impedance biosensor.

**Fig. 6:**
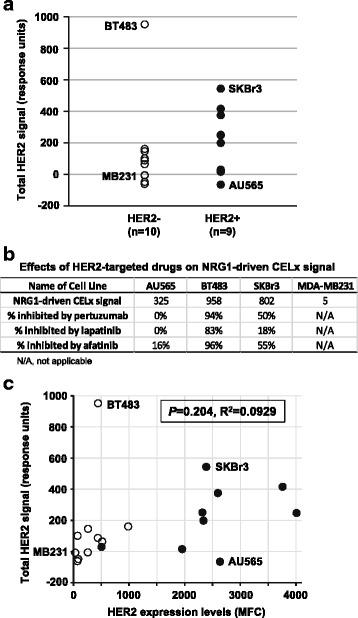
CELx HSF Test signals in HER2+ and HER2- breast cancer cell lines. **a** HER2+ cell lines (*n =* 9) and HER2- cell lines (*n =* 10) were evaluated with the CELx HSF test as described in the Methods. The sum of NRG1b- and EGF-driven HER2 signals that can be inhibited by the HER2-specific mAb pertuzumab was approximated as response units for all cell lines and plotted. **b** Comparison of NRG1b-driven CELx signals in AU565, BT483, SKBr3 (HER2+ reference cell line), and MDA-MB231 (HER2- reference cell line) and sensitivities to HER2-targeted drugs (pertuzumab, lapatinib, and afatinib). **c** HER2 expression levels in HER2+ (*n =* 9) and HER2- cell lines (*n =* 10) were determined by fluorescence flow cytometry (mean fluorescence channel units, MFC) and plotted against the corresponding HER2 signal determined by CELx HSF test (response units) for each cell line. No correlation between the two parameters was observed (*P* = 0.204, R^2^ = 0.0929). *Empty circles*, HER2- cell lines; *Filled circles*, HER2+ cell lines. The locations of BT483, AU565, SKBr3 (HER2+ reference cell line) and MDA-MB231 (HER2- reference cell line) are indicated

The CELx HSF test was used to determine the amount of HER2 participation in NRG1b- and EGF-driven activity in the HER2+ (*n =* 9) and HER2- (*n =* 10) breast cancer cell lines in the presence and absence of pertuzumab. EGF and NRG1b are both capable of initiating signaling of HER family homodimers and heterodimers without HER2 participation. The antibody pertuzumab’s mechanism of action for disruption of ligand induced signaling is by binding to HER2 and prevention of HER2 dimerization with other HER family members. When pertuzumab was applied to the different cell samples, the results showed different levels of attenuation of EGF and NRG1b signals depending on the cell line. The variable attenuation by pertuzumab is related to the amount of HER2 participation in each growth factor initiated signaling for each of the different cell samples. Thus pertuzumab is an appropriate tool for the determination of HER2 participation in signaling activity measured by the CELx HSF Test and was used for subsequent data analyses. Results for ligand-driven HER2 CELx signals from all HER2+ and HER2– cell lines are presented in Fig. 6a. In this plot, the sum of NRG1b- and EGF-driven HER2 signals that can be inhibited by pertuzumab in the same CELx HSF test was used to calculate the net CELx HSF test value (an indicator of HER2 signaling activity) for each cell line, as described in the Methods. Overall, the average CELx HSF values were higher in the HER2+ group (mean 224 ± 203 response units, range = −65 to 544) than in the HER2- group (mean 139 ± 296 response units, range = −61 to 952). However, there were cell lines from both groups, which produced similar signaling activities in CELx HSF tests. For example, BT483, a HER2- cell line, had one of the highest levels of HER2 signaling activity (~1000 response units) (Fig. 6a) that was more consistent with the highest HER2+ group. Conversely, there were HER2+ cell lines, such as AU565, that displayed a very low level of HER2 signaling and were more similar to the lowest HER2- group. Based on this dataset, 5 out of 9 (56.6%) HER2+ cell lines and 1 out of 10 (10%) HER2- cell lines had high CELx HSF values (>224 response units, the average of the HER2+ group), which may be considered indicative of potentially abnormally high HER2 pathway signaling activity.

As further confirmation of the CELx HSF test results for AU565 and BT483, their responses to pertuzumab and lapatinib were evaluated. The evaluation focused on data for NRG1b-driven signaling with these drugs given the results showing the primary importance of this mechanism in HER2 signaling. NRG1b-driven CELx signals and sensitivities to these drugs are presented in Fig. 6b. The HER2+ cell line, AU565, had high a NRG1-driven signal, but was insensitive to either pertuzumab or lapatinib. This indicated that despite the high HER2 expression level, HER2 was not involved in the NRG1b-driven signaling, and thus AU565 cells were not sensitive to the drug designed to block HER2 activity in the CELx test. This finding is consistent with the previous finding that AU565 was insensitive to lapatinib [36]. In contrast, the HER2- cell line, BT483, which was found to have a very high NRG1-driven signal, was highly sensitive to pertuzumab and treatment resulted in nearly complete CELx test signal attenuation. This result indicated that HER2 participated greatly in NRG1b-driven signaling, although HER2 expression is low in BT483. Thus, as expected, BT483 was also sensitive to lapatinib. The effect of lapatinib was reinforced by CELx test signal suppression results with afatinib (Gilotrif) [37], an irreversible covalent kinase inhibitor of all ErbB-family members with intrinsic catalytic activity, including HER1, HER2, and HER4 (Fig. 6b). Afatinib also inhibits HER3 transphosphorylation. Collectively, these findings suggest that the CELx HSF test may be a more sensitive and specific indicator of HER2 pathway activity than methods currently used to determine HER2 expression status. Furthermore, correlation analysis results showed that HER2 protein expression levels were not significantly correlated with HER2 signaling amplitudes determined by the CELx HSF test (Fig. 6c) (*P* = 0.204, R^2^ = 0.0929), which further supports the conclusion that HER2 pathway activity can be independent of HER2 expression status.

**Fig. 7:**
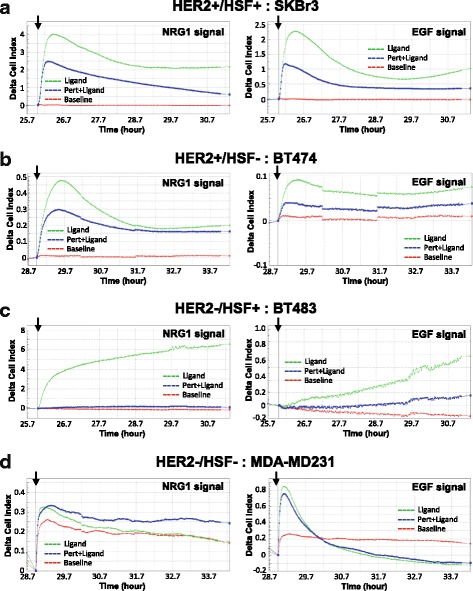
Subtypes of CELx HSF curves. Representative CELx time-course curves representing HER2+/HSF+ (HER2+ cells having high HER2 signaling activities) (**a**), HER2+/HSF- (HER2+ cells having low HER2 signaling activities) (**b**), HER2-/HSF+ (HER2- cells having high HER2 signaling activities) (**c**), and HER2-/HSF- (HER2- cells having low HER2 signaling activities) (**d**) are shown. For display purposes, NRG1b and EGF-driven HER2 CELx signals are shown in separated panels. CELx curves are displayed using Delta CI values to demonstrate the relative signals to the time point (*arrow*) when the stimulus (EGF or NRG1b) was added. *Red* curves, unstimulated cells (control); *Green* curves, cells stimulated with ligand (NRG1b or EGF); *Blue* curves, cells stimulated with ligand in the presence of drug (pertuzumab)

Overall, when comparing the magnitude of HER2 ligand driven signaling activities determined by CELx HSF tests, there existed at least four subtypes of cell lines, including HER2+/HSF+ (HER2+ cells having high HER2 signaling activities), HER2+/HSF- (HER2+ cells having low HER2 signaling activities), HER2-/HSF+ (HER2- cells having high HER2 signaling activities), and HER2-/HSF- (HER2- cells having low HER2 signaling activities). The CELx curves characteristic of each subtype are shown in Fig. 7.

### Application of the CELx HSF test to evaluate dynamic HER2 signaling function in patient samples *ex vivo*

**Fig. 8:**
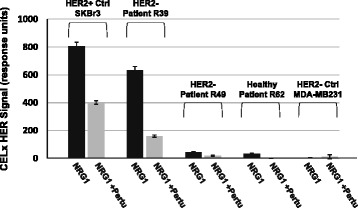
Validation of CELx HSF test in patient tissue specimen-derived primary cells *ex vivo*. Primary epithelial cells derived from two HER2- (R39 and R49) patients with breast cancer and one healthy control subject (R62) were subjected to CELx HSF tests. Responses of NRG1b-driven HER2 CELx signals with and without pertuzumab for these primary cells are plotted along with those for the HER2+ reference cell line (SKBr3) and the HER2- reference cell line (MDA-MB231) as bar charts. *Black bars*, cells stimulated with NRG1b; *Grey bars*, cells stimulated with NRG1b in the presence of pertuzumab. HER2- Patient R39 has approximately 80% of the NRG1 CELx ﻿signal of HER2+ cell line SKBr3

Following initial results with well-established reference cell lines, CELx HSF tests were applied to primary epithelial cells derived from two patients with breast cancer and one healthy control subject as further proof-of-concept. Typical flow cytometry results for primary samples in short-term, zero passage culture confirmed a heterogeneous population of myo and luminal epithelial cells and low stromal cell content (Additional file 7: Figure S5). The responses from primary cells are presented in Fig. 8 of NRG1b-driven HER2 CELx signals with and without pertuzumab. The results show that primary cells from a HER2– breast cancer patient (R39) displayed an amplified CELx HSF signal due to HER2 participation that was in the range of the HER2+ reference cell line SKBr3, whereas primary cells from another patient with HER2– breast cancer (R49) and a healthy subject (R62) had CELx HSF signals similar to the HER2- reference cell line MDA-MB231. These results demonstrate that the CELx HSF test can be applied to generate high-content temporal data reflecting the dynamic status of HER2 signaling network in patient tumor-derived primary cells. The test revealed very different HER2 pathway signaling activity in samples R39 and R49 despite both being classified as HER2– based on clinical HER2 expression status.

**Fig. 9:**
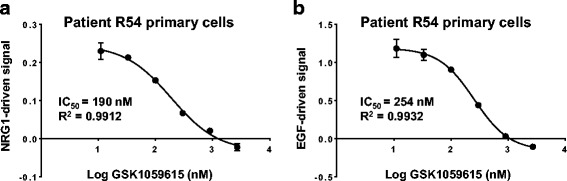
The PI3K/AKT pathway significantly contributes to the ligand-driven HER2 signaling activities detected by CELx HSF tests in patient-derived breast tumor primary cells. Patient R54 breast tumor-derived primary cells (15,000 cells per well) pre-seeded in sensor plates were treated with a serial titration of GSK1059615 (0 nM to 2700 nM) two hours prior to stimulation with NRG1b (800 pM) (**a**) or EGF (600 pM) (**b**). The dose-dependent inhibitory effect of GSK1059615 on ligand-driven HER2 signals is shown

To further corroborate the findings from the pathway deconvolution experiments in breast cancer cell lines, the results with patient-derived breast tumor primary cells (R54) further demonstrated that fresh patient-derived cells could produce sufficient signal upon stimulation, confirming the pathway deconvolution results in a more physiologic setting (Fig. 9). Consistent with the test results in breast cancer cell lines, both EGF and NRG1b-driven HER2 signals from primary R54 cells were detected by CELx HSF tests and were dependent on PI3K activation (Fig. 9) but not by MAPK activation (Additional file 8: Figure S6 demonstrated with﻿ lack of activity of trametinib, inhibitor of MEK1/2 in MAPK pathway) for this primary pathway dysfunctional specimen.

## Discussion

Accurate determination of HER2 status is critical for optimizing use of HER2-targeted therapies and improving therapeutic outcomes. Existing HER2 tests (either IHC or FISH) [38] only provide information on HER2 protein expression or gene amplification and do not provide data on the functional status of the HER2 protein and its signaling network. By definition, these tests exclude HER2- breast cancer patients for treatment with HER2 targeted therapies who may benefit from them. This study demonstrates the feasibility of the CELx HSF test, a label-free impedance-based live cell assay, which quantifies HER2 functional signaling pathway activity in response to HER2 agonists and antagonists in a real-time manner.

Breast cancer cell lines have been widely used as model systems for studies on breast cancer pathobiology and new therapy development [39–41]. Neve et al. reported that the recurrent genomic and transcriptional characteristics of 51 breast cancer cell lines mirror those of 145 primary breast tumors [39]. The present study successfully employs HER2+ and HER2- breast cancer cell lines in optimization, characterization, and analytical specificity and sensitivity verification studies during the course of development of a novel functional signaling test. This work includes the IHC HER2+ clinical reference 3+ cell line SKBr3. We demonstrate that breast cancer cell lines and primary cells share many similarities regarding the phenotypic alterations (cell adhesion and temporal patterns) in response to HER family pathway agonists and antagonists when measured by CELx.

Following the cell line work, three different samples of primary cells were analyzed to demonstrate the feasibility of applying the CELx HSF test to clinical specimens. For the clinical specimen, FACS data first established that cultured primary cells derived from fresh patient tumor tissue were of the epithelial type with stromal content typically 5% or less. Several biomarkers that define luminal and basal types of epithelial cells were used [42]. The tumors maintained multiple phenotypically distinct subsets (see Additional file 7: Figure S5) of epithelial cells during the culture period.

### Defining and measuring receptor function using the CELx HSF test

Reliability, analytical specificity, sensitivity, and accuracy are essential prerequisites for the CELx HSF test to be considered for clinical diagnostic applications. When performing label-free biosensor-based viable cell assays, complexity is inherent and caution was exercised to test whether the signal was limited to a biological response resulting from a single molecule type binding to a single receptor type effecting signaling on a single pathway. In this study, a series of experiments were performed to demonstrate the selectivity and specificity of the assay for cell lines and primary cells.

FDA-approved HER2 inhibitors that treat HER2-positive breast cancer in clinical settings were used in this study to serve three purposes. First, the inhibitors helped to identify the specificity of the impedance signal arising from treatment of the cells with growth factors. Second, the anti-HER2 mAb inhibitors isolated the impedance signal arising solely due to HER2 participation in the growth factor activation of HER family pathway signaling. This provides a level of detail regarding the specificity of the selected reagents by using antagonists that work most proximal to signal initiation, receptor dimerization and receptor tyrosine kinase priming, thereby most effectively defining HER2 participation and isolating early signaling events before signal branching takes place. Finally, previous studies suggest differential sensitivities to the HER2 inhibitors among the cells lines used here [34]. Thus, the utilization of these HER2 signaling inhibitors would help to define the potential correlation of CELx signal with drug sensitivity in these cell lines.

The data for testing baseline effect of pertuzumab or lapatinib alone on cells (Fig. 3, Panel a) indicate that neither have significant effect on SKBr3 cells in an HER2 overexpressing cell line. The same results were found when other HER2-overexpressing cell lines were tested and this result is in good agreement with published data indicating these drugs are cytostatic, not cytotoxic, and only slow cell passage through G_1_ [43, 44].

Trastuzumab was not selected for evaluation in this study because its primary mechanism of action, as reported by its manufacturer, is not HER2-driven signaling inhibition, but instead antibody-dependent cell-mediated cytotoxicity (ADCC). Any results studying the effect of trastuzumab on HER2-driven signaling would thus be confounded by the lack of direct linkage between the activity we are measuring, HER2 signaling, and trastuzumab’s primary mechanism of action (ADCC). Since the CELx HSF Test is designed to assess HER2 participation in HER family signaling, pertuzumab, a known HER2 dimer blocker, was selected instead to confirm the amount of HER2 participation in HER family signaling in this assay.

All HER2 CELx signals tested are agonist- and antagonist-concentration dependent within physiological doses in the picomolar to nanomolar range. When a HER2 antagonist (e.g. pertuzumab or lapatinib) is added with agonist, the cells show a significantly attenuated delta CI compared to the signal for addition of agonist only, indicative of a blocked HER2 signaling response. The work employs carefully selected components that have known specificity and well characterized affinity at concentrations that reduce the likelihood of activation of other pathways from high concentrations of agonists. EGF and NRG1b are very specific ligands for HER1 and HER3 receptors. Multiple literature references cite *in vitro* receptor affinity of ~100pM for EGF and NRG1b [45, 46]. This is in close agreement with the CELx test data presented here and in line with the concentrations that have been selected to measure agonism and antagonism in the CELx test.

Further dissection of the information from rich CELx data suggests sources of NRG1-driven test signal that is linked to more than just HER2/HER3 heterodimerization. In the SKBr3, HER2+ cells (Fig. 3), lapatinib was able to reduce the NRG1 and EGF stimulation signals nearly to zero while pertuzumab was only able to attain partial (<50%) attenuation of the NRG1 and EGF-induced signals. The pertuzumab result indicates that HER2 was only partly involved as a heterodimer with HER1 and HER3 in the NRG1 and EGF stimulations and the remaining NRG1 and EGF signal could be indicated primarily for homodimer activity at HER1 and HER3, respectively. The lapatinib result on EGF stimulation of HER1 seems to confirm this. However, the lapatinib result on NRG1 signal cannot be explained quite as simply because HER3 is reported to possess only weak kinase activity and thus may be unable to generate very large signals [47, 48]. This opens the possibility that HER3 binds NRG1 and heterodimerizes with HER1 or other receptor tyrosine kinases [49] to activate and sustain PI3K signaling or that HER3 expression is upregulated and its dephosphorylation is stalled; both are mechanisms that have been described previously [50, 51]. This result highlights the difficulty of making limited protein time point analyses to determine drug efficacy and points to the value of a functional activity test such as the CELx HSF.

### Determining pathway involvement

Next, a determination was made that the HER2-associated downstream signaling pathways controlling the cellular responses were quantified by the CELx HSF test. A series of pathway deconvolution experiments were performed using specific agonists and antagonists of different pathway members. The MAPK and the PI3K/AKT pathways are the two major pathways downstream of all HER family receptors [10]. Ligand binding, receptor phosphorylation, and receptor-intrinsic kinase activation in normal cells leads to the propagation of signals that regulate important cellular processes such as cell adhesion, migration, proliferation, and survival [10]. The present study focuses on PI3K and MAPK pathways and dissects the signaling mechanistically related to the HER2-driven phenotypic alterations. In both breast cancer cell lines and primary cancer cells, the data show that PI3K, not MAPK, is the downstream effector that contributes most significantly to the ligand-driven HER2 signal in the CELx HSF test for these cancer cell samples. This finding suggests that HER2 heterodimers, especially HER2/HER3, that form as determined by the use of a HER2 dimer blocker, are probably dominant in these types of breast tumors. The findings from the current study are in agreement with the existing literature, which suggests a high level of PI3K signaling in a subset of breast tumors and that HER2/HER3 is a strong driver of oncogenic HER2 signaling through PI3K activation in this subset [12–15].

The PI3K pathway is a highly complex signal progression model even though the pathway is often described in terms that imply otherwise. Multiple positive and negative effector proteins and mechanisms of PI3K pathway function and dysfunction have been demonstrated to attenuate and direct inhibition of PI3K activity in different patients. For example, mutations of PI3K combined with copy number variants or RAS activation and heritable cell-to-cell variability can affect the efficacy of inhibitors [52–54]. Therefore, it is not unexpected that incomplete response to PI3K inhibition would be seen in different patients. GSK1059615 on breast cancer cell lines *in vitro* inhibits the phosphorylation of Akt at S473, with an IC50 of 40 nM [55], which translates well to the cellular IC_50_ potency we find for the compound’s attenuation of signaling.

The CELx HSF test detects unexpected signaling and drug sensitivity in a HER2- breast cancer cell line. BT-483 is defined as having a PI3K activating mutation, E542K [52, 56]. This activating mutation has been reported to act as a resistance mechanism [57] to HER2 signal inhibitors in HER2 overexpressed cell lines, which is speculated to explain the mutation’s correlation with poor prognosis. Despite having only normal expression levels of HER2 receptor, BT-483 recorded very high levels of NRG1b initiated PI3K initiated activity that was almost completely inhibited by pertuzumab and lapatinib. In fact, BT-483’s HER2-driven signaling activity was higher than activity found in all of the HER2+ cell lines evaluated (Fig. 6). This finding suggests a more complex role for PI3K mutation as a resistance mechanism for HER2 signal inhibition. Other HER2-negative cell lines tested in this study, such as MCF-7 and MDA-MB-361 also have similar (E545K) PI3K activating mutations. However, the HER2-driven signaling test measured in these cell lines was consistent with normal pathway activity. This suggests that the high NRG1b initiated PI3K activity in BT-483 cells is not related solely to this PI3k mutation.

Endpoint cell-based assays provide a one-time “snapshot” of a focused biological event (e.g., phosphorylation of HER2 at a single time point). Although protein or gene based assays provide incremental information, they are still classical endpoint assays that reflect only the relative activity of a limited set of proteins that may be involved in disease propagation, and the results do not describe the dynamics or real-time status of the complete HER2 signaling network in a particular patient. Given that infinite permutations of circumstances are present and each persons’ genomic or proteomic status does not yet describe the *in vivo* nature of that individual’s disease, a truly functional dynamic analysis may be more appropriate. Furthermore, allosterism, differential transient phosphorylation, signaling crosstalk, and a myriad of mechanisms of drug effect may contribute to the quantitative and qualitative activity of the HER2 signaling pathway in any particular patient [15, 58, 59]. Mylona et al. report on opposing effects of multisite phosphorylation shaping a signaling protein response to activation [60]. They conclude that their “results challenge the common assumption that multisite modification events act unidirectionally and can only be reversed or limited by antagonistic enzymes such as phosphatases.” The Mylona et al. study brings into doubt what is already suspected about the utility of correlations built upon single time point, limited site protein phosphorylation analyses’ for assessment of pathway function in whole cells. Santarpia et al. review biomarker studies in breast cancer and conclude: “It is likely that it is the combined effect of all genomic variations that drives the clinical behavior of a given cancer [61]. Furthermore, entirely new classes of oncogenic events are being discovered in the noncoding areas of the genome and in noncoding RNA species driven by errors in RNA editing. In light of this complexity, it is not unexpected that, with the exception of HER2 amplification, no robust molecular predictors of benefit from targeted therapies have been identified.” These factors contribute to the difficulty in using a protein quantification readout to comprehensively quantify signaling pathway regulation that relates drug response and therapeutic outcome prediction [15, 58, 59].

To verify the CELx HSF test concept, HER2+ (*n =* 9) and HER2- (*n =* 10) breast cancer reference cell lines were chosen. Fluorescence flow cytometry measurement of HER2 protein expression levels demonstrated HER2 expression data largely consistent with published CCLE data on *HER2* gene copy number in these cell lines [34]. However, the HER2 signal function determined by CELx HSF tests did not show any correlation with HER2 expression levels in these cell lines. The CCLE database documents HER2+ cell lines that are not responsive to HER2-targeted drugs *in vitro*. Recent retrospective analyses of previous clinical trials indicated that there is no significant correlation between *HER2* gene copy number or total HER2 protein and clinical benefit from trastuzumab [6, 7], although the molecular basis remains unclear and could be very diverse amongst patients. The results obtained from the present study suggest that some HER2+ breast tumors may not respond to HER2-signal inhibitors because they do not actually exhibit increased HER2 signaling activity or functional dependence on HER2 signaling, whereas some HER2- breast tumors could benefit from HER2-signal inhibitors because the HER2 pathway is abnormally active in these tumors. Collectively, the present data strongly suggests that HER2 signaling pathway dysfunction is the critical prerequisite for determining whether tumor cells respond to HER2-signal inhibitors.

The present test seeks to identify HER2-negative samples that have abnormally overactive HER2 signaling. Previous work by others have presented results that describe elevated protein ligands [62] of the HER family as the most likely cause of the PI3K activation in HER2 negative patients. The CELx test results with exogenous ligand equally applied to all samples suggest that there are other more systemic causes besides abundance of ligand. Other published work proposes elevated HER3 expression in HER2-negative cancers as leading to abnormal signaling in HER2 negative patients [8]. Several authors propose increased expression of HER2 in cancer stem cells to explain HER2– patient abnormal signaling or responsiveness to HER2-targeted therapy [63, 64]. The flow cytometry data presented here do not support any of these receptor overexpression mechanisms.

Taken together, the results in this study demonstrate that the CELx HSF test is a selective and specific assay for monitoring the dynamic cellular pathway signaling status in live cells in response to ligand–receptor interactions and between receptors and receptor-targeting drugs. Functional assessment of HER2 signaling in live tumor cells with the CELx HSF test represents a possible new approach to diagnosing HER2-driven cancer in individual patients who have normal HER2 expression levels. It is envisioned that this test would be deployed in a central lab, where patient tumor specimens would be delivered and tested. To be successful, greater than 80–90% of clinical specimens must yield test results. To further develop this method, analytical validation studies meeting CAP (College of American Pathologists) and CLIA (Clinical Laboratory Improvement Amendments) established guidelines for Laboratory Developed Tests would be required. Finally, the clinical validity of using HER2-driven signaling activity as a diagnostic biomarker must be confirmed in a clinical trial that evaluates whether HER2-breast cancer patients with abnormal HER2-driven signaling benefit from treatment with HER2 signal inhibitors.

## Conclusions

HER2 receptor levels do not correlate with the functional activity measured by the CELx test. The wide range of HER2-driven signaling levels measured suggests it may be possible to make a distinction between normal and abnormal levels of activity. Measurement of HER2 signaling activity in the tumor cells of breast cancer patients is a feasible approach to explore as a biomarker with the CELx test to identify HER2-driven cancers not currently diagnosable with IHC or genomic techniques. Analytical validation studies and clinical trials treating HER2- patients with abnormal HER2-driven signaling would be required to evaluate the analytical and clinical validity of using this functional biomarker as a diagnostic test to select patients for treatment with HER2 targeted therapy.

## Additional files

Additional file 1:
**Table S1.** Antibodies used in this study. All epitopes were extracellular with the exceptions of ER and PR. All antibodies were purchased from companies as listed who provided empirical demonstration of each of the antibodies for our applications. (DOCX 30 kb)
Additional file 2:
**Figure S1.** The PI3K/AKT pathway significantly contributes to the ligand-driven HER2 signaling activities detected by CELx HSF tests. (A and B) SKBr3 cells were seeded in sensor plates and then treated with a serial titration of the PI3K/AKT pathway inhibitor GDC-0941 (0 nM to 2700 nM) two hours prior to maximal stimulation with NRG1b (800 pM) (A) or EGF (600 pM) (B). CELx curves are displayed using Baseline Delta CI values. The relative CELx signals were baseline subtracted to the time point (*arrow*) when the stimulus (EGF or NRG1b) was added and the signals induced by stimulus alone without the drug (GDC-0941) were set as baselines. Dose–response curves of GDC-0941 inhibition on NRG1b and EGF-driven HER2 signals are shown in the insets. (PDF 92 kb)
Additional file 3:
**Figure S2.** The MEK/ERK pathway does not significantly contribute to the ligand-driven HER2 signaling activities detected by CELx HSF tests. SKBr3 cells were seeded in sensor plates and then treated with a serial titration of the MEK/ERK pathway inhibitor trametinib (0 nM to 2700 nM) two hours prior to maximal stimulation with NRG1b (800 pM) (A) or EGF (600 pM) (B). CELx curves are displayed using Delta CI values to demonstrate the relative signals to the time point (*arrow*) when the stimulus (EGF or NRG1b) was added. No trametinib dose-dependent inhibition on NRG1b or EGF-driven HER2 signals was detected (*insets*). (PDF 107 kb)
Additional file 4:
**Figure S3.** The p38 MAPK pathway does not significantly contribute to the ligand-driven HER2 signaling activities detected by CELx HSF tests. SKBr3 cells were seeded in sensor plates and then treated with a serial titration of the p38 MAPK pathway inhibitor doramapimod (0 nM to 810 nM) two hours prior to maximal stimulation with NRG1b (800 pM) (A) or EGF (600 pM) (B). CELx curves are displayed using Delta CI values to demonstrate the relative signals to the time point (*arrow*) when the stimulus (EGF or NRG1b) was added. No doramapimod dose-dependent inhibition on NRG1b or EGF-driven HER2 signals was detected. (PDF 99 kb)
Additional file 5:
**Figure S4.** The JNK pathway does not significantly contribute to the ligand-driven HER2 signaling activities detected by CELx HSF tests. SKBr3 cells were seeded in sensor plates and then treated with a serial titration of the JNK pathway inhibitor SP600125 (0 nM to 2700 nM) two hours prior to maximal stimulation with NRG1b (800 pM) (A) or EGF (600 pM) (B). CELx curves are displayed using Delta CI values to demonstrate the relative signals to the time point (*arrow*) when the stimulus (EGF or NRG1b) was added. No SP600125 dose-dependent inhibition on NRG1b or EGF-driven HER2 signals was detected. (PDF 98 kb)
Additional file 6:
**Table S2.** Comparison of HER2 levels in HER2+ and HER2- breast cancer cell lines. Data from this study, determined by FACS and expressed in mean fluorescence channel units (MFC); Data from CCLE database [34], expressed in Log2.(DOCX 34 kb)
Additional file 7:
**Figure S5.** Fluorescence flow cytometry showing two epithelial markers that delineate basal epithelial, stromal (fibroblast), progenitor epithelial, and luminal cells in primary cell R49 from short term culture. The image shows very few fibroblasts and significant luminal, basal and some progenitor populations. In contrast, a second image (*right panel*) is shown for the combined experimental runs of SKBr3 (luminal breast cancer reference) and MDA-MB-231 (basal epithelial breast cancer reference) cell lines demonstrating their more monoclonal character. (PDF 179 kb)
Additional file 8:
**Figure S6.** The MAPK pathway does not significantly contribute to the ligand-driven HER2 signaling activities detected by CELx HSF tests in breast cancer primary cells. Patient R54 breast tumor-derived primary cells (15,000 cells per well) pre-seeded in sensor plates were treated with a serial titration of the MEK/ERK pathway inhibitor trametinib (0 nM to 810 nM) two hours prior to stimulation with NRG1b (800 pM) (A) or EGF (600 pM) (B). No trametinib dose-dependent inhibition on NRG1b or EGF-driven HER2 signals was detected when data were subjected to dose–response inhibitory curve fitting. (PDF 44 kb)

## Abbreviations

CAP: College of American Pathologists
CELx HSF Test: CELx™ HER2 Signaling Function test
CI: Cell index
CLIA: Clinical Laboratory Improvement Amendments
ECM: Extracellular matrix
EGF: Epidermal growth factor
EGFR or HER1: EGF receptor or human epidermal growth factor receptor 1
ER: Estrogen receptor
HER2-: HER2 negative or HER2 low or HER2 normal
HER2: Human epidermal growth factor receptor 2
HER2+: HER2 positive or HER2 overexpressed or HER2 gene amplified
HTS: High-throughput screening
IGF-1: Insulin like growth factor-1
IHC: Immunohistochemistry
ISH: *In situ* hybridization
NRG1: Neuregulin 1
RTCA: Real time cell analyzer
