# Supplementary material for: Development of a test that measures real-time HER2 signaling function in live breast cancer cell lines and primary cells
Source: BMC Cancer. 2017 Mar 16;17:199. doi: 10.1186/s12885-017-3181-0 (PMC5356237; doi:10.1186/s12885-017-3181-0)
Supplement: Additional file 1: — Table S1. Antibodies used in this study. All epitopes were extracellular with the exceptions of ER and PR. All antibodies were purchased from companies as listed who provided empirical demonstration of each of the antibodies for our applications. (DOCX 30 kb) [file 12885_2017_3181_MOESM1_ESM.docx]

| **Description of Antibody** | **Vendor** |
| --- | --- |
| mouse anti-human HER2-phycoerythrin (PE), clone 24D2 | Biolegend, San Diego, CA |
| mouse anti-human HER1 conjugated with AlexaFluor 647, clone EGFR.1 | BD Biosciences, San Jose, CA |
| mouse anti-human HER3 conjugated with PerCP-sFluor710, clone SGP1 | eBioscience, San Diego, CA |
| mouse anti-human EPCAM conjugated with AlexaFluor 488, clone MH99 | eBioscience, San Diego, CA |
| mouse anti-human Claudin4 conjugated to PE, clone 382321 | R&D Systems Minneapolis, MN |
| rat anti-human CD49f conjugated to PerCP/eFluor710, clone eBioGoH3 | eBioscience, San Diego, CA |
| mouse anti-human CD10 conjugated to Allophycocyanin (AP), cloneHL10a | BioLegend, San Diego, CA |
| rabbit polyclonal anti-human estrogen receptor alpha (ERα) conjugated to AlexaFluor488 | Bioss, Woburn, MA |
| mouse anti-human progesterone receptor (PRG) conjugated to eFluor660, clone KMC912 | eBioscience, San Diego, CA |

**Supplemental Table 1. Antibodies used in this study**

*All epitopes were extracellular with the exceptions of ER and PR. All antibodies were purchased from companies as listed who provided empirical demonstration of each of the antibodies for our applications.
