# Supplementary figures and images for: Development of a test that measures real-time HER2 signaling function in live breast cancer cell lines and primary cells
Source: BMC Cancer. 2017 Mar 16;17:199. doi: 10.1186/s12885-017-3181-0 (PMC5356237; doi:10.1186/s12885-017-3181-0)

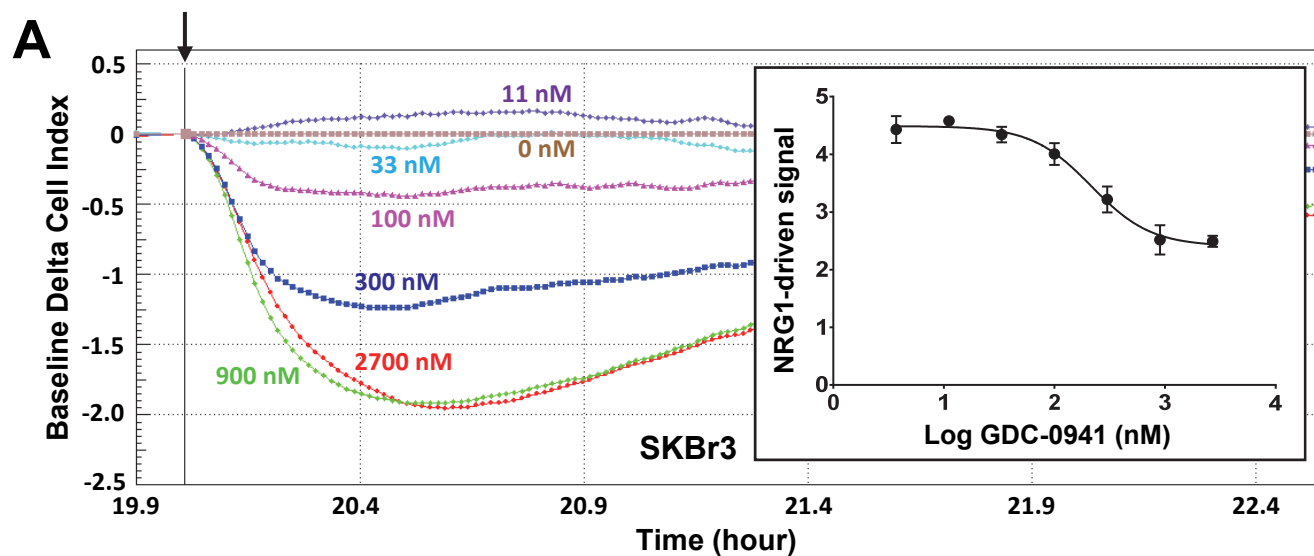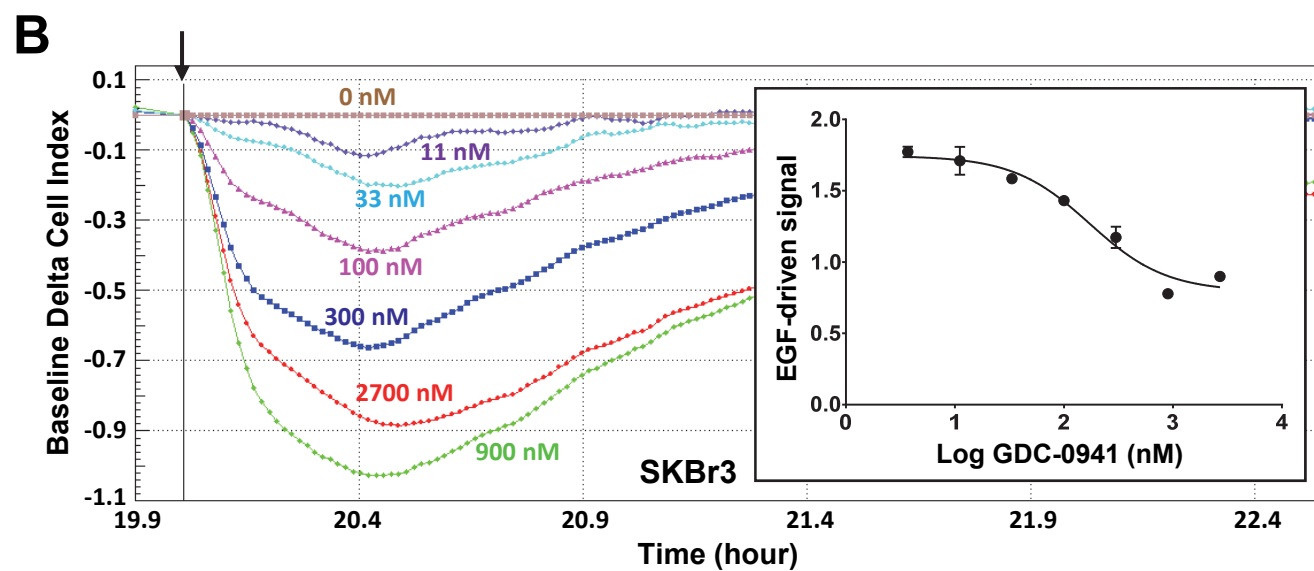

Supplement: Additional file 2: — Figure S1. The PI3K/AKT pathway significantly contributes to the ligand-driven HER2 signaling activities detected by CELx HSF tests. (A and B) SKBr3 cells were seeded in sensor plates and then treated with a serial titration of the PI3K/AKT pathway inhibitor GDC-0941 (0 nM to 2700 nM) two hours prior to maximal stimulation with NRG1b (800 pM) (A) or EGF (600 pM) (B). CELx curves are displayed using Baseline Delta CI values. The relative CELx signals were baseline subtracted to the time point (arrow) when the stimulus (EGF or NRG1b) was added and the signals induced by stimulus alone without the drug (GDC-0941) were set as baselines. Dose–response curves of GDC-0941 inhibition on NRG1b and EGF-driven HER2 signals are shown in the insets. (PDF 92 kb) [file 12885_2017_3181_MOESM2_ESM.pdf]

**A**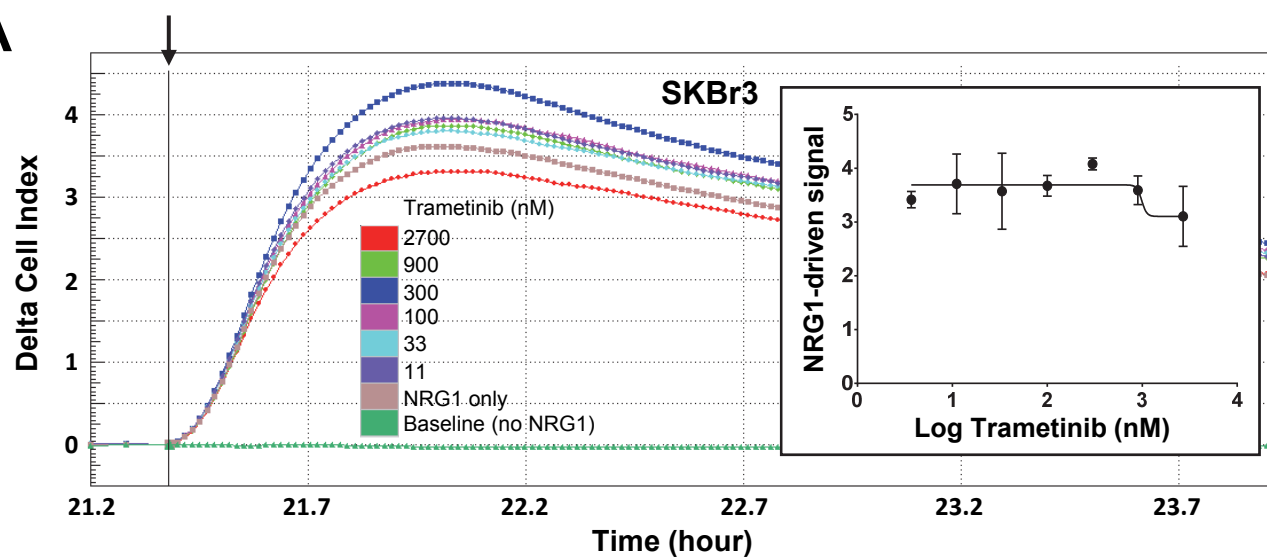**B**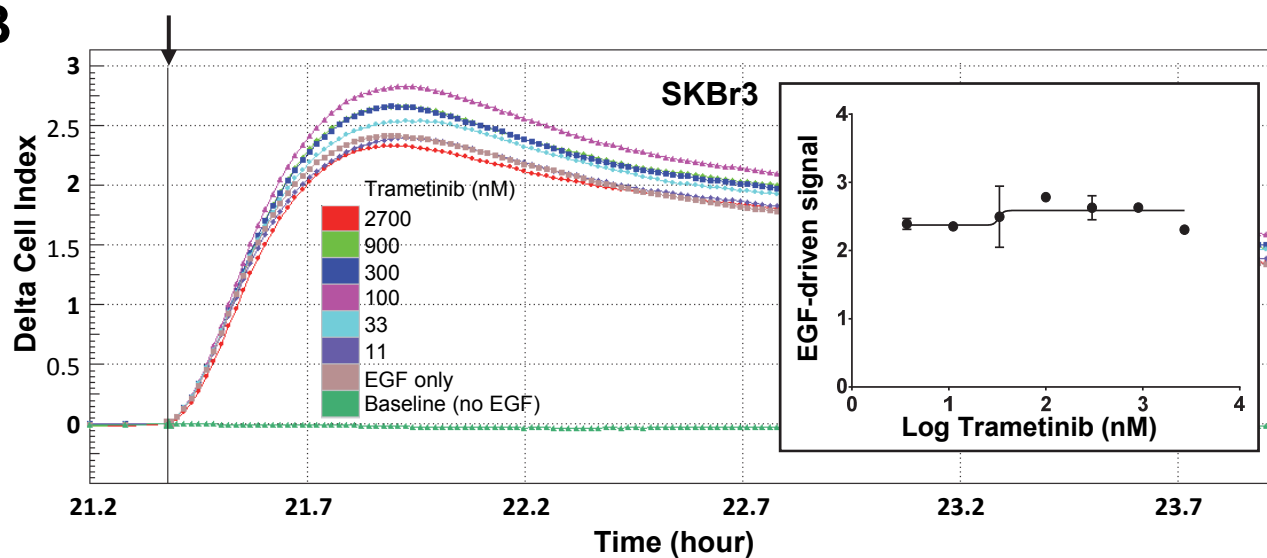

Supplement: Additional file 3: — Figure S2. The MEK/ERK pathway does not significantly contribute to the ligand-driven HER2 signaling activities detected by CELx HSF tests. SKBr3 cells were seeded in sensor plates and then treated with a serial titration of the MEK/ERK pathway inhibitor trametinib (0 nM to 2700 nM) two hours prior to maximal stimulation with NRG1b (800 pM) (A) or EGF (600 pM) (B). CELx curves are displayed using Delta CI values to demonstrate the relative signals to the time point (arrow) when the stimulus (EGF or NRG1b) was added. No trametinib dose-dependent inhibition on NRG1b or EGF-driven HER2 signals was detected (insets). (PDF 107 kb) [file 12885_2017_3181_MOESM3_ESM.pdf]

**A**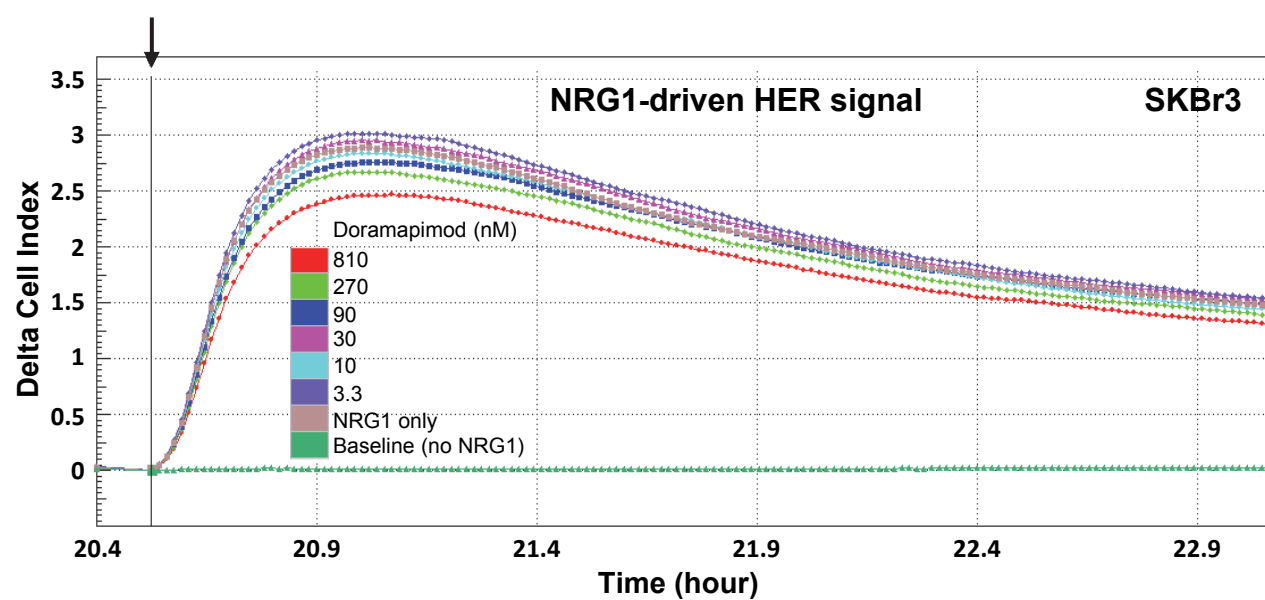**B**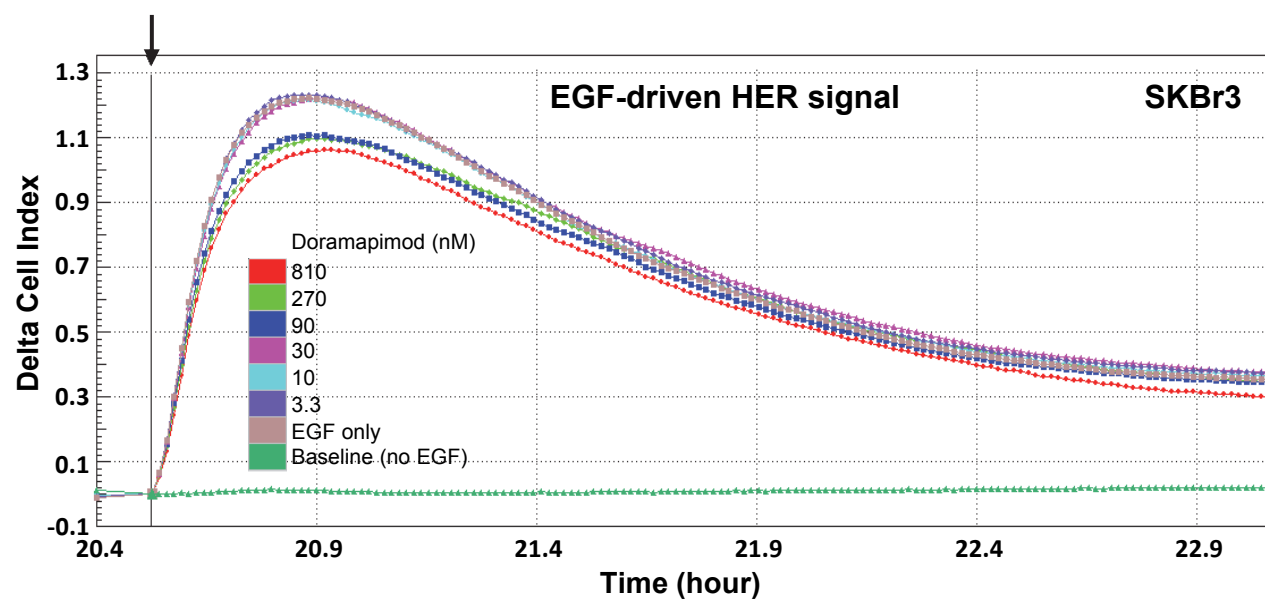

Supplement: Additional file 4: — Figure S3. The p38 MAPK pathway does not significantly contribute to the ligand-driven HER2 signaling activities detected by CELx HSF tests. SKBr3 cells were seeded in sensor plates and then treated with a serial titration of the p38 MAPK pathway inhibitor doramapimod (0 nM to 810 nM) two hours prior to maximal stimulation with NRG1b (800 pM) (A) or EGF (600 pM) (B). CELx curves are displayed using Delta CI values to demonstrate the relative signals to the time point (arrow) when the stimulus (EGF or NRG1b) was added. No doramapimod dose-dependent inhibition on NRG1b or EGF-driven HER2 signals was detected. (PDF 99 kb) [file 12885_2017_3181_MOESM4_ESM.pdf]

**A**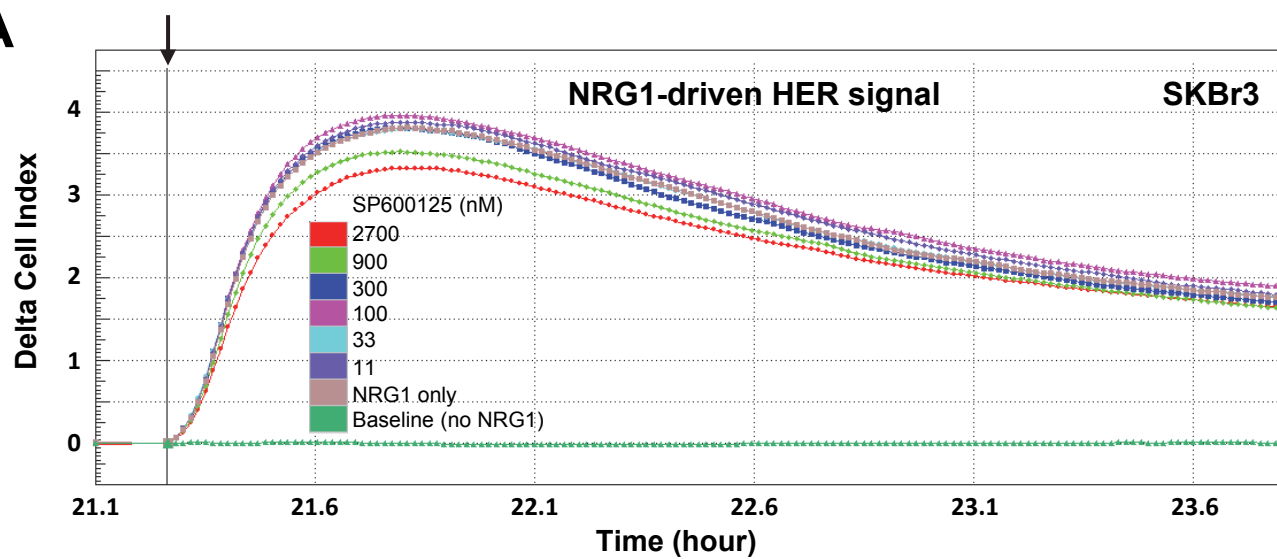**B**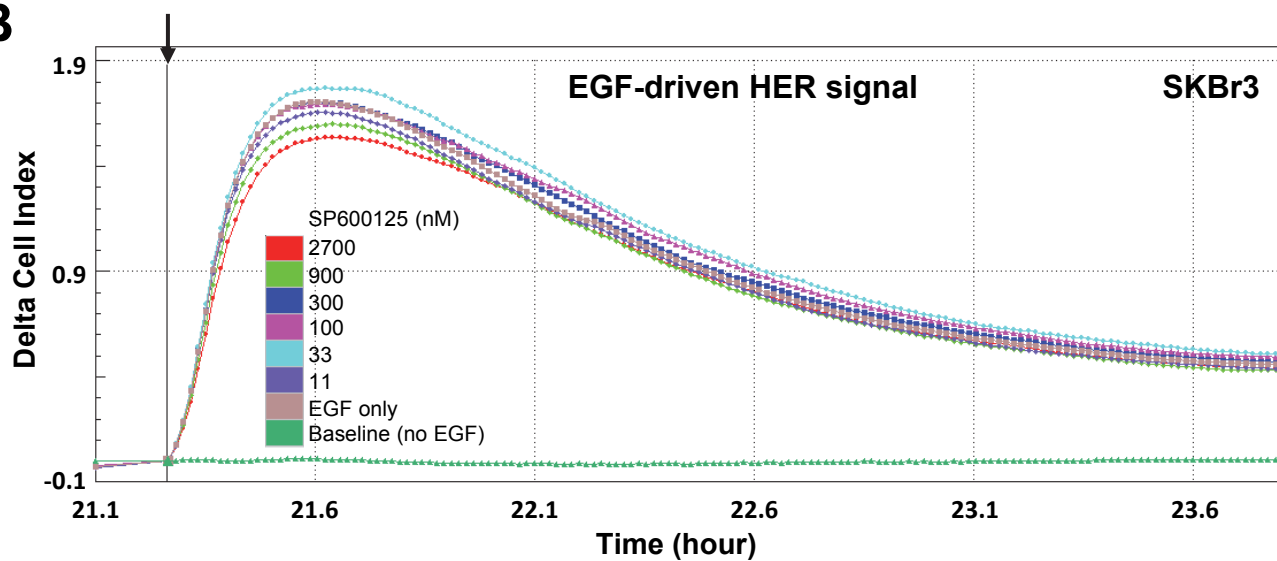

Supplement: Additional file 5: — Figure S4. The JNK pathway does not significantly contribute to the ligand-driven HER2 signaling activities detected by CELx HSF tests. SKBr3 cells were seeded in sensor plates and then treated with a serial titration of the JNK pathway inhibitor SP600125 (0 nM to 2700 nM) two hours prior to maximal stimulation with NRG1b (800 pM) (A) or EGF (600 pM) (B). CELx curves are displayed using Delta CI values to demonstrate the relative signals to the time point (arrow) when the stimulus (EGF or NRG1b) was added. No SP600125 dose-dependent inhibition on NRG1b or EGF-driven HER2 signals was detected. (PDF 98 kb) [file 12885_2017_3181_MOESM5_ESM.pdf]

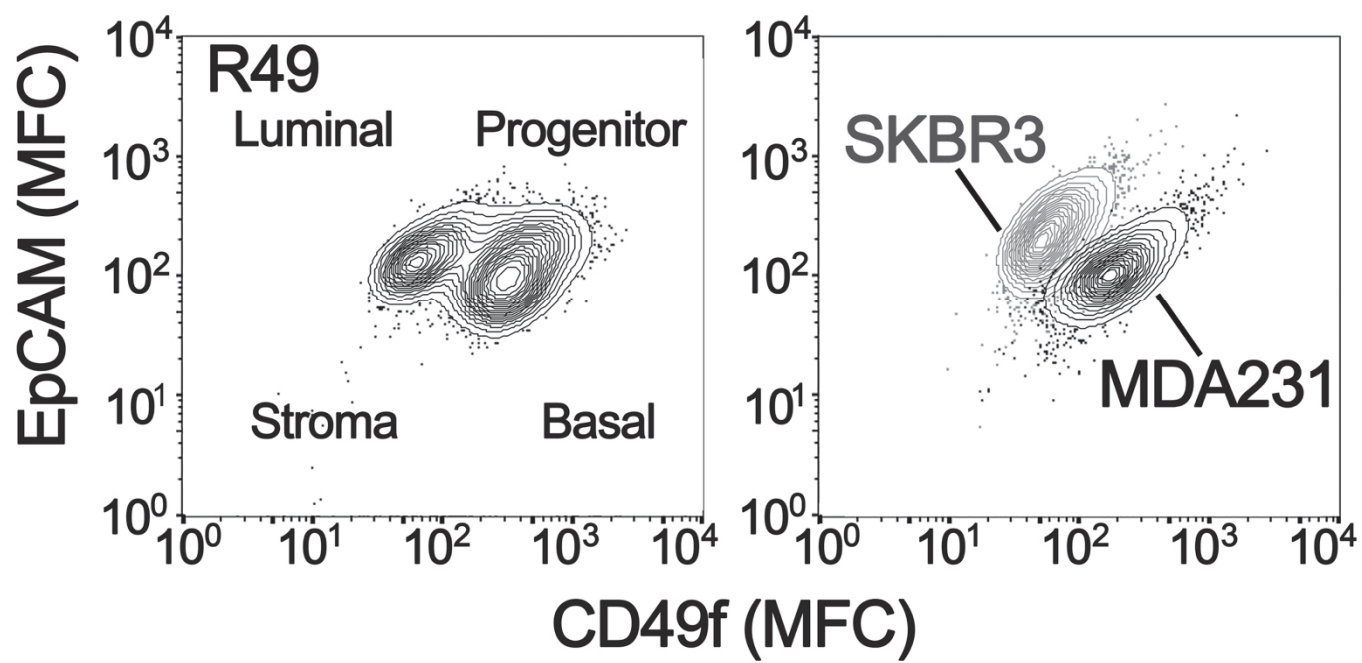

Supplement: Additional file 7: — Figure S5. Fluorescence flow cytometry showing two epithelial markers that delineate basal epithelial, stromal (fibroblast), progenitor epithelial, and luminal cells in primary cell R49 from short term culture. The image shows very few fibroblasts and significant luminal, basal and some progenitor populations. In contrast, a second image (right panel) is shown for the combined experimental runs of SKBr3 (luminal breast cancer reference) and MDA-MB-231 (basal epithelial breast cancer reference) cell lines demonstrating their more monoclonal character. (PDF 179 kb) [file 12885_2017_3181_MOESM7_ESM.pdf]

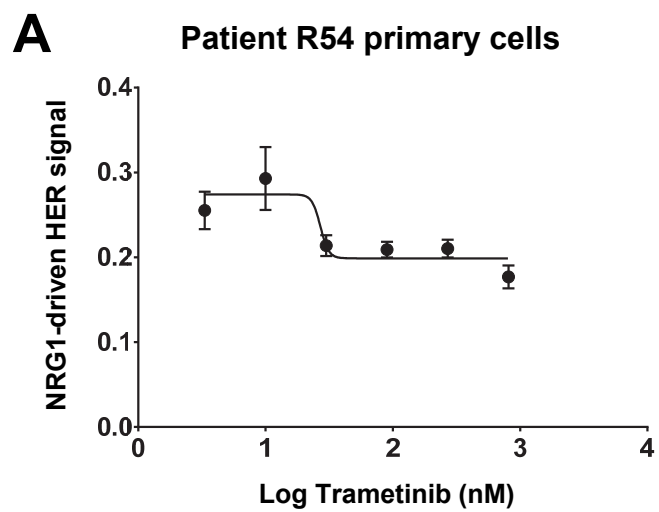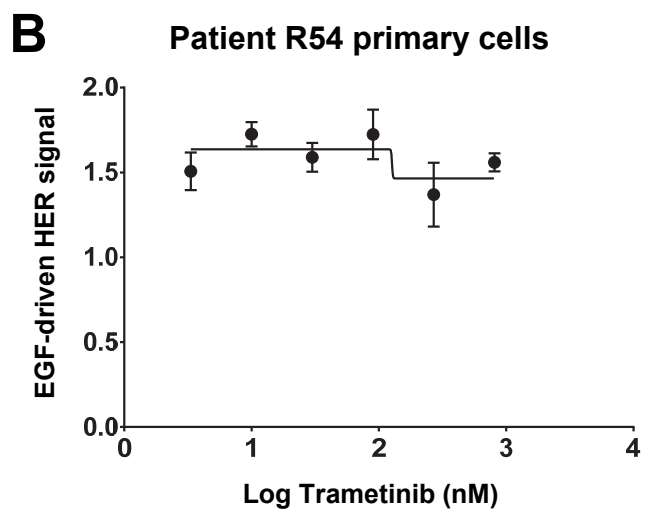

Supplement: Additional file 8: — Figure S6. The MAPK pathway does not significantly contribute to the ligand-driven HER2 signaling activities detected by CELx HSF tests in breast cancer primary cells. Patient R54 breast tumor-derived primary cells (15,000 cells per well) pre-seeded in sensor plates were treated with a serial titration of the MEK/ERK pathway inhibitor trametinib (0 nM to 810 nM) two hours prior to stimulation with NRG1b (800 pM) (A) or EGF (600 pM) (B). No trametinib dose-dependent inhibition on NRG1b or EGF-driven HER2 signals was detected when data were subjected to dose–response inhibitory curve fitting. (PDF 44 kb) [file 12885_2017_3181_MOESM8_ESM.pdf]
