# Supplementary material for: Development of a test that measures real-time HER2 signaling function in live breast cancer cell lines and primary cells
Source: BMC Cancer. 2017 Mar 16;17:199. doi: 10.1186/s12885-017-3181-0 (PMC5356237; doi:10.1186/s12885-017-3181-0)
Supplement: Additional file 6: — Table S2. Comparison of HER2 levels in HER2+ and HER2- breast cancer cell lines. Data from this study, determined by FACS and expressed in mean fluorescence channel units (MFC); Data from CCLE database [34], expressed in Log2.(DOCX 34 kb) [file 12885_2017_3181_MOESM6_ESM.docx]

**Supplemental Table 2. Comparison of HER2 levels in HER2+ and HER2- breast cancer cell lines.**

|  | **HER2 Protein Level (MFC) ^1^** | ***HER2* mRNA Level (Log2) ^2^** | ***HER2* Gene Copy (Log2) ^2^** |
| --- | --- | --- | --- |
| **HER2+ Cell Lines** |  |  |  |
| HCC1954 | 4009 | 12.6 | 3.46 |
| EFM192A | 3758 | 13.1 | 2.72 |
| AU565 | 2633 | 13.0 | 2.71 |
| ZR75-30 | 2595 | 12.9 | 2.74 |
| HCC202 | 2316 | 12.2 | 2.45 |
| HCC1569 | 2335 | 12.1 | 3.07 |
| SKBR3 | 2386 | 12.0 | 2.78 |
| BT474 | 1953 | 11.8 | 3.96 |
| MDA-MB361 | 505 | 11.0 | 2.22 |
| **HER2- Cell Lines** |  |  |  |
| MDA-MB453 | 517 | 10.1 | 1.16 |
| MDA-MB175vii | 438 | 9.7 | 0.06 |
| BT483 | 441 | 9.6 | 0.17 |
| ZR75-1 | 261 | 8.6 | -0.09 |
| T47D | 72 | 8.2 | 0.51 |
| CAMA1 | 257 | 7.9 | 0.15 |
| MCF7 | 86 | 7.9 | -0.97 |
| MDA-MB415 | 75 | 7.4 | -0.09 |
| HCC1428 | 984 | 7.0 | -0.68 |
| MDA-MB231 | 32 | 6.6 | 0.09 |
| MDA-MB134vi | 16 | 6.6 | -0.12 |

^1^ Data from this study, determined by FACS and expressed in mean fluorescence channel units (MFC);

^2^ Data from CCLE database [34], expressed in Log2.
